# The Kampo Medicine Rokumigan Possesses Antibiofilm, Anti-Inflammatory, and Wound Healing Properties

**DOI:** 10.1155/2014/436206

**Published:** 2014-04-30

**Authors:** James Liao, Jabrane Azelmat, Lei Zhao, Masami Yoshioka, Daisuke Hinode, Daniel Grenier

**Affiliations:** ^1^Oral Ecology Research Group, Faculty of Dentistry, Laval University, 2420 Rue de la Terrasse, Quebec City, QC, Canada G1V 0A6; ^2^Department of Periodontics, West China Hospital of Stomatology, Sichuan University, Chengdu, Sichuan 610041, China; ^3^Department of Oral Health Science and Social Welfare, Institute of Health Biosciences, The University of Tokushima Graduate School, 18-15 Kuramotocho 3-Chome, Tokushima 770-8504, Japan; ^4^Department of Hygiene and Oral Health Science, Institute of Health Biosciences, The University of Tokushima Graduate School, 18-15 Kuramotocho 3-Chome, Tokushima 770-8504, Japan

## Abstract

Periodontal diseases, which are inflammatory diseases of bacterial origin affecting the tooth-supporting tissues, are characterized by inflammation and destruction of gingival connective tissue and alveolar bone, and may lead to tooth loss. The aim of the study was to investigate Rokumigan, a Kampo Japanese traditional medicine made of six different plants, for its capacity to prevent biofilm formation by *Fusobacterium nucleatum*, to inhibit interleukin-6 (IL-6) and interleukin-8 (IL-8) secretion by mucosal cells, and to promote wound healing in a fibroblast model. Using a microplate colorimetric assay, Rokumigan prevented biofilm formation by *F. nucleatum*, while it had no effect on bacterial growth. Rokumigan inhibited IL-6 secretion in both epithelial cells and fibroblasts stimulated with lipopolysaccharide. However, it caused no significant inhibition of IL-8 secretion by both cell types. Rokumigan significantly increased proliferation and migration of gingival fibroblasts in a wound healing assay. In conclusion, the Kampo formulation Rokumigan, through suppression of biofilm formation by *F. nucleatum*, inhibition of IL-6 secretion by gingival epithelial cells and fibroblasts, and promotion of wound healing in a fibroblast model, may have potential application for periodontal diseases.

## 1. Introduction

Periodontal diseases are inflammatory disorders of the gingival tissues and can be broadly categorized into gingivitis and periodontitis. More specifically, chronic periodontitis is characterized by inflammation of the tooth-supporting tissues, bone resorption, and ultimately tooth loss. While a majority of adults suffer from mild to moderate periodontitis, approximately 5% to 20% of any population is affected by severe, generalized periodontitis [1]. Although additional clinical trials involving greater numbers of study patients are required, considerable evidence has been brought that correlates chronic periodontitis with systemic complications such as cardiovascular diseases, preterm baby delivery, and rheumatoid arthritis [2, 3]. Chronic periodontitis has two etiological components: (i) the presence of specific bacterial species, called periodontopathogens and mostly Gram-negative, colonizing the subgingival sites as a biofilm [4], and (ii) the host immune response to these pathogens that results in the production of high levels of inflammatory mediators and matrix metalloproteinases (MMPs) [5, 6].

The Gram-negative anaerobic bacterium* Fusobacterium nucleatum* increases in numbers in subgingival sites of periodontitis patients [7]. This periodontopathogen plays a central role in biofilm formation since it is known to serve as a bridge organism being able to interact with both early and late colonizers as well as Gram-negative and Gram-positive bacteria [8]. Moreover,* F. nucleatum* has been shown to adhere to and invade oral epithelial cells [9].

Fibroblasts and epithelial cells are major constituents of the gingival tissues and act as a physical barrier to prevent invasion by periodontopathogens [10]. In response to stimulation by periodontopathogens, these cells can secrete proinflammatory cytokines such as interleukin-6 (IL-6) and interleukin-8 (IL-8) [11, 12]. Conventional treatment for periodontitis involves mechanical removal of the subgingival biofilm, which induces the host immunodestructive response. In severe cases, antibiotics and surgical treatments are necessary to prevent further loss of periodontal tissues. Given the critical role of the biofilm-induced inflammatory response in disease progression and severity, compounds with the ability to suppress biofilm formation and/or to modulate the host immune response are of high interest for periodontal therapy.

Kampos are Japanese derivatives of traditional Chinese medicine (TCM) and have been used to treat a wide variety of conditions for hundreds of years [13]. Today, 148 Kampo formulations are regulated by the Japanese national health insurance system and prescribed by licensed medical professionals as well as authorized Kampo practitioners [13, 14]. Despite that, few studies have been conducted to elucidate the medicinal properties and mechanisms of action of Kampos. Typical Kampos are made of five to nine herbs; while leaves and seeds are occasionally used, herb roots or rhizomes are most commonly used. One of the advantages of using a mixture of different herbs, as found in Kampos, relates to the fact that there is a greater variety of bioactive compounds as compared to what is found in a single herb. These molecules may interact with one another in complex ways resulting in synergistic effects which can produce enhanced or even novel effects compared to those of each individual plant extract. In a previous study, we reported that Kampos containing rhubarb may be of interest for controlling periodontal disease through inhibition of* P. gingivalis* growth and virulence properties [15]. As a continuation of our ongoing studies aimed to identify beneficial properties of Kampos for periodontal disease, in this study, we investigated Rokumigan (TJ-87) for its capacity to prevent biofilm formation by* F. nucleatum*, to inhibit IL-6 and IL-8 secretion by mucosal cells, and to promote wound healing in a fibroblast model.

## 2. Materials and Methods

### 2.1. Compounds

Rokumigan (TJ-87) was obtained from Tsumura Co., Ltd. (Tokyo, Japan), as packaged pellets. This Kampo is produced by combining six crude herbal extracts (Table 1). Rokumigan stock solution was prepared by adding 2 mg of pellets to 1 mL of distilled water. The mixture was heated to 37°C for 1 h and filtered through a 0.22 *μ*m pore size membrane.

### 2.2. Biofilm Formation by* F. nucleatum*

A 24 h culture of* F. nucleatum* ATCC 25586 in Todd-Hewitt broth (BBL Microbiology Systems, Cockeysville, MD, USA) was diluted in fresh broth medium to obtain an optical density at 660 nm (OD_660_) of 0.2. Samples (100 *μ*L) were added to the wells of a 96-well tissue culture plate containing 100 *μ*L of serial dilutions (400 to 50 *μ*g mL^−1^) of Rokumigan in broth medium. Control wells with no compounds or no bacteria were also prepared. After incubation for 48 h at 37°C under anaerobic conditions (N_2_/H_2_/CO_2_ :  80/10/10), the OD_660_ was recorded, and spent media and free-floating bacteria were removed by aspiration. The wells were washed three times with distilled water and the* F. nucleatum* biofilms were stained with 0.04% crystal violet (100 *μ*L) for 15 min. The wells were washed four times with 50 mM phosphate-buffered saline pH 7.2 (PBS) to remove unbound crystal violet dye and dried for 2 h at 37°C. After adding 100 *μ*L of 95% (v/v) ethanol to each well, the plate was shaken for 10 min to release the stain from the biofilms and the absorbance at 550 nm (A_550_) was recorded.

### 2.3. Epithelial Cell and Fibroblast Culture Conditions

The immortalized human gingival epithelial cell line OBA-9 [16], which was kindly provided by* M. Mayer *(Department of Microbiology, Institute of Biomedical Sciences, University of São Paulo, São Paulo, Brazil), was cultured in keratinocyte serum-free medium (K-SFM; Life Technologies Inc., Burlington, ON, Canada), containing insulin, epidermal growth factor, fibroblast growth factor, and 100 *μ*g mL^−1^ of penicillin G/streptomycin. The primary human gingival fibroblast cell line HGF-1 (ATCC CRL-2014) was purchased from the American Type Culture Collection (ATCC) (Manassas, VA, USA). HGF-1 were cultured in Dulbecco's modified Eagle's medium (DMEM) supplemented with 4 mM L-glutamine (HyClone Laboratories, Logan, UT), 10% heat-inactivated fetal bovine serum (FBS), and 100 *μ*g mL^−1^ of penicillin G-streptomycin. Both cell lines were incubated at 37°C in a 5% CO_2_ atmosphere until they reached confluence.

### 2.4. Inflammatory Cytokine Secretion

Gingival epithelial cells and fibroblasts were seeded (500 *μ*L) in 12-well tissue culture plates at 1 × 10^6^ cells mL^−1^. The cells were treated with decreasing concentrations of the Rokumigan (100 to 25 *μ*g mL^−1^) and were incubated at 37°C in 5% CO_2_ for 2 h prior to being stimulated with 1 *μ*g mL^−1^ of* Aggregatibacter actinomycetemcomitans* (ATCC 29522) lipopolysaccharide (LPS). The LPS was isolated according to a previously published procedure [17]. After a 24 h incubation, the culture medium supernatants were collected and stored at −20°C until being used. Cells incubated in culture medium with or without Rokumigan but not stimulated with LPS were used as controls. Commercial enzyme-linked immunosorbent assay (ELISA) kits (eBioscience, San Diego, CA, USA) were used to quantify IL-6 and IL-8 concentrations in the cell-free culture supernatants according to the manufacturer's protocols. The rated sensitivities of the ELISA kits were 2 pg mL^−1^.

### 2.5. Wound Healing Assay

The ability of Rokumigan to promote wound healing was assessed using the Oris Pro Cell Migration Assay kit (Platypus Technologies, Madison, WI, USA) according to the manufacturer's protocol. Briefly, 5 × 10^4^ gingival fibroblasts in DMEM-0.5% FBS (200 *μ*L) were seeded into wells of a 96-well microplate. The type I collagen-coated wells were equipped with in-place stoppers to create a migration area on the bottom of each well. The stoppers were removed after an overnight incubation, the medium was aspirated, and fresh DMEM-0.5% FBS with different concentrations of Rokumigan (400 to 25 *μ*g mL^−1^) was added. After an incubation of 3 d, the cells were washed twice with sterile PBS and stained with 5 *μ*M calcein acetoxymethyl ester. Cell migration was quantified using a microplate reader (485 nm/528 nm: excitation/emission wavelengths) with a black bottom mask. An* Aloe vera* extract (400 *μ*g mL^−1^; Chromadex, Irvine, CA) was used as positive control.

### 2.6. Statistical Analysis

Data were expressed as the means ± standard deviations of triplicate assays from at least two independent experiments. Differences between the means were analyzed for statistical significance using Student's *t*-test and were considered significant at *P* < 0.05.

## 3. Results

**Figure 1:**
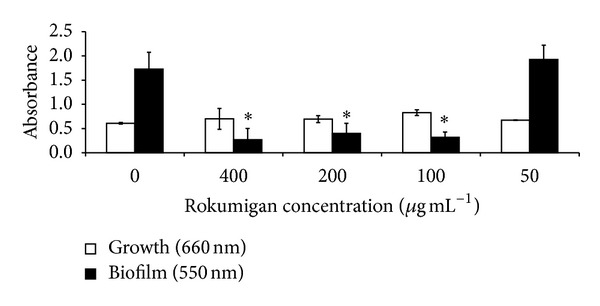
Effects of Rokumigan on* F. nucleatum *growth and biofilm formation. ∗Significant inhibition at *P* < 0.05 using Student's *t*-test.

The ability of Rokumigan to interfere with growth and biofilm formation of* F. nucleatum *was first evaluated. As reported in Figure 1, Rokumigan did not show any effect on growth of* F. nucleatum*. However, at concentrations ≥100 *μ*g mL^−1^, it suppressed significantly the formation of biofilm by* F. nucleatum* (Figure 1).

**Figure 2:**
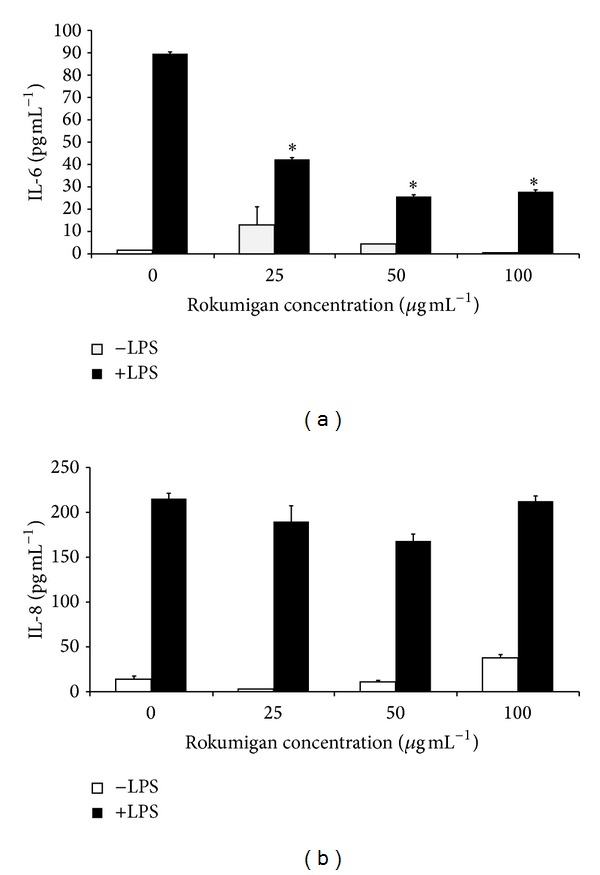
Effects of Rokumigan on IL-6 (a) and IL-8 (b) secretion by human gingival fibroblasts (HGF-1). ∗Significant inhibition at *P* < 0.05 using Student's *t*-test.

**Figure 3:**
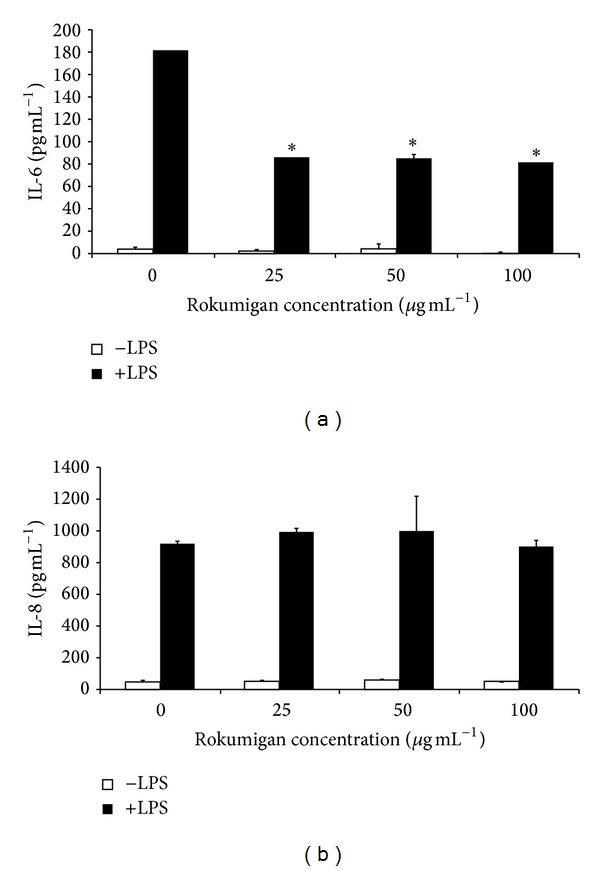
Effects of Rokumigan on IL-6 (a) and IL-8 (b) production by human gingival epithelial cells (OBA-9). ∗Significant inhibition at *P* < 0.05 using Student's *t*-test.

Rokumigan was then tested for its ability to inhibit IL-6 and IL-8 secretion by gingival fibroblasts and epithelial cells. LPS stimulation of gingival fibroblasts increased IL-6 secretion by 67-fold and IL-8 secretion by 15-fold (Figure 2). LPS stimulation of epithelial cells increased IL-6 secretion by 42-fold and IL-8 secretion by 18-fold (Figure 3). Rokumigan promoted very slightly IL-6 or IL-8 secretion at any of the concentrations tested (Figures 2 and 3). Rokumigan significantly decreased the secretion of IL-6 by both epithelial cells and fibroblasts stimulated with LPS (Figures 2 and 3). At up to 100 *μ*g mL^−1^, the inhibitory effect was more important in gingival fibroblasts. More specifically, at 100 *μ*g mL^−1^, Rokumigan decreased IL-6 secretion in fibroblasts and epithelial cells by 71% and 55%, respectively. Rokumigan exhibited no significant inhibitory effect on IL-8 secretion by LPS-stimulated fibroblasts and epithelial cells at any of the concentrations tested (25–100 *μ*g mL^−1^).

**Figure 4:**
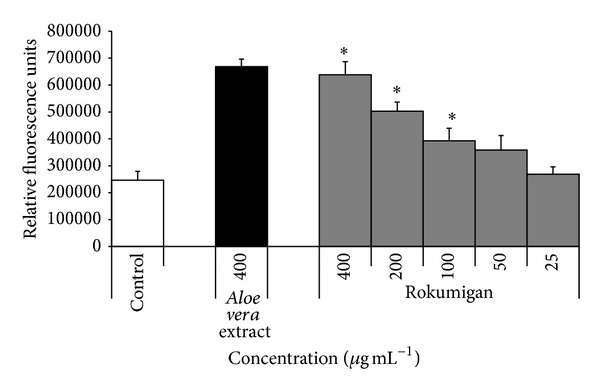
Effects of Rokumigan on fibroblast wound healing. An extract of* A. vera* was used as positive control. ∗Significant increase at *P* < 0.05 using Student's *t*-test.

We then used a fibroblast migration assay to investigate whether Rokumigan can promote wound healing. Rokumigan increased significantly the migration of gingival fibroblasts when added at a concentration ≥100 *μ*g mL^−1^ (Figure 4). At 400 *μ*g mL^−1^, the highest concentration tested, it increased fibroblast migration by 158%. The effect of Rokumigan on fibroblast migration was comparable to the effect observed with the* Aloe vera* extract used as a positive control (Figure 4).

## 4. Discussion

Kampo medicines prescribed by medical practitioners offering alternatives to western medications have been integrated into the Japanese health care system about 50 years ago [13, 14]. Kampo formulations are made of several plant extracts and consequently, the large variety of phytochemicals they contain is likely to act synergistically to provide their beneficial effects. In the present study, we identified new properties associated with Rokumigan (TJ-87) that support the potential of this Kampo for periodontal health. Rokumigan, which is made of six different herbs (Table 1), is one of the most common herbal formulas used for body enrichment, kidney ailments, and edema [18]. Moreover, this Kampo has been reported to possess antioxidant and free radical scavenging activities [19].

Biofilms, which are defined as structured microbial communities attached to surfaces, play a critical role in most bacterial infections in humans. In the oral cavity, biofilms allow bacteria to evade immune defenses and to better resist to mechanical removal and chemotherapeutic agents. Our study showed that Rokumigan can prevent biofilm formation by* F. nucleatum*, a periodontopathogen that acts as bridging bacteria between early and late colonizers. The mechanism responsible for inhibition of biofilm formation may involve a modification of the cell surface hydrophobicity or neutralization of adhesins involved in bacterial aggregation. The inhibition of biofilm formation is an attractive target for the development of new therapies in the prevention of bacterial infections, particularly infections of mucosal surfaces. The fact that Rokumigan acts by preventing bacterial adhesion rather than by inhibiting growth may represent an advantage since bacteria are unlikely to develop resistance.

The host immune response to periodontopathogens, which results in the release of inflammatory mediators by host mucosal and immune cells, is known to mediate localized tissue destruction in periodontitis [20]. The periodontal tissues and gingival crevicular fluid of periodontitis patients contain high levels of inflammatory mediators, and their etiological correlation with periodontitis has been clearly demonstrated [20]. It is thus logical to investigate therapeutic approaches that modulate the host response in order to manage chronic periodontitis. Recently, two Kampo medicines, Shosaikoto (TJ-9) and Orento (TJ-120), were reported to possess anti-inflammatory activity by their ability to suppress LPS-induced prostaglandin E_2_ (PGE_2_) production by gingival fibroblasts [21, 22]. In the present study, we showed that Rokumigan can inhibit LPS-induced IL-6 secretion by both gingival fibroblasts and epithelial cells. Interestingly, IL-6 expression was found to be higher at sites of periodontal inflammation and closely related to clinical severity of periodontitis [23]. In addition, its levels increase in the diseased gingiva of patients with periodontitis compared to periodontally healthy subjects [24]. IL-6 is a multifunctional cytokine that can induce osteoclast formation and promote bone resorption [25, 26]. While this occurs naturally as part of normal bone remodeling, an excess of IL-6 production, as found during periodontitis, leads to the destruction of alveolar bone. By attenuating IL-6 secretion, Rokumigan may thus contribute to reducing bone resorption. To further support the potential of Rokumigan for reducing bone resorption, Shim et al. [27] brought evidence that* Yukmijihwang-tang *(Chinese equivalent of Rokumigan) may have therapeutic potential in bone diseases by preventing osteoclast differentiation and inhibiting the bone-resorptive activity of differentiated osteoclasts. While Rokumigan inhibited IL-6 secretion in epithelial cells and fibroblasts, it did not significantly attenuate IL-8 secretion. This suggests that the signaling pathways involved in LPS-modulated IL-6 and IL-8 secretion are different and that Rokumigan specifically acts on the one related to IL-6 release.

Wound healing is a complex process involving cell attachment to various components of the extracellular matrix as well as cell migration and proliferation [28]. During wound healing, fibroblasts play a critical role in forming healing (granulation) tissue by proliferating, migrating, and remodeling the extracellular matrix by the* de novo* synthesis of matrix molecules [28]. Since fibroblasts are critical for the healing of gingival wounds, studying the effects of Rokumigan on this cell type will help to determine whether this Kampo contributes to the gingival wound healing process. We showed that Rokumigan has a positive effect in a wound healing model by enhancing dose-dependently fibroblast migration using the Oris Cell Migration Assay kit. These effects may be of interest for the treatment and/or management of gingival wounds where fibroblasts are the major cells and are mainly responsible for healing following the treatment of periodontal diseases. It is unknown at this time whether the wound healing effect is due to an individual ingredient within Rokumigan or a synergistic combination of two or more ingredients. Further research into the effects of Rokumigan and its individual ingredients is necessary.

To prevent periodontal disease progression, mechanical procedures are used to remove the dental biofilm. Although these procedures are effective in managing the majority of periodontitis patients, there are situations in which conventional therapy does not always achieve the desired clinical outcome. Control of disease in individuals with significantly increased risk for periodontitis (smokers, diabetics, or individuals possessing genetic predisposition) or who do not respond to conventional therapy may require adjunctive treatments. In this regard, the Kampo formulation Rokumigan, which possesses multiple beneficial properties, may represent a promising tool for controlling periodontal diseases.

## 5. Conclusion

Periodontal diseases are multifactorial diseases and, as such, have many potential targets for therapy. In the present study, we showed that Rokumigan, a Kampo Japanese traditional medicine, can act on both components of the etiology of periodontitis: bacterial biofilm and host inflammatory response. In addition, Rokumigan promoted wound healing in a fibroblast model. These properties support the fact that Rokumigan may be considered as a promising adjunctive therapy for periodontal disease.

## Figures and Tables

**Table 1:** Herbal composition of Rokumigan (TJ-87).

Herb	Common name	Scientific name	Amount (per 20 g)
Rehmanniae Radix	Chinese fox glove root	*Rehmannia glutinosa* Liboschitz var. *purpurea * Makino	5 g
Dioscoreae Rhizoma	Korea yam	*Dioscorea batatas* Decaisne	3 g
Corni Fructus	Asiatic cornelian cherry fruit	*Cornus officinalis* Siebold et Zuccarini	3 g
Hoelen	Poria	*Poria cocos* Wolf	3 g
Moutan Cortex	Peony tree root cortex	*Paeonia suffruticosa* Andrews	3 g
Alismatis Rhizoma	Water plantain root	*Alisma orientale* Juzepczuk	3 g
